# The Story of the Insane from Year to Year

**Published:** 1901-05-25

**Authors:** 


### THE STORY OF THE INSANE FROM YEAR TO YEAR.

(THIRTEENTH SERIES.)

### WORCESTER COUNTY ASYLUM.

THIS asylum contained 1,130 patients on January 1st, 1899, and 1,146 on December 31st. There were 210 first admissions, 15 re-admissions, 87 recoveries, 11 discharged relieved, 20 not improved, and 91 died. The average number resident during the year was 1,137, and the total number of persons under care was 1,355. The recoveries stand at 40·5 per cent. of the admissions, and the deaths at 8 per cent. of the average number resident. Of the deaths 20 were caused by cerebral and spinal diseases, 21 by phthisis, 11 by pneumonia, 2 by pleurisy, 1 by bronchitis, and 7 by colitis and enteritis. The general death-rate was not high, but consumption accounted foı 23 per cent. of the total deaths. Dr. Braine-Hartnell thinks the crowded state of the wards accounts for the high mortality from this disease, and he, like so many other medical superintendents, says that separate wards are required for its treatment. Doubtless a proper sanatorium for this purpose would be highly desirable, and as the Worcester Asylum is full, this would be good opportunity for building one. A pavilion for the treatment of phthisical cases would be as well, or better, built of wood than of any other material; it could be finished in a month or two, and it would to some extent relieve the pressure for accommodation. Scarlet fever and typhoid both made their appearance, and, what is really worse than either, colitis broke out. It is curious to notice how often this troublesome disease manifests itself in a great many asylums, and how little is yet known of its origin. The committee report that the medical superintendent and the assistant medical officers satisfactorily discharge their duties. The weekly charge to the unions has been reduced from 7s. 10d. to 7s. 7d. per head per week.

